# Quality assessment of clinical practice guidelines for neonatal sepsis using the Appraisal of Guidelines for Research and Evaluation (AGREE) II Instrument: A systematic review of neonatal guidelines

**DOI:** 10.3389/fped.2022.891572

**Published:** 2022-08-16

**Authors:** Yasser S. Amer, Lana A. Shaiba, Adnan Hadid, Jasim Anabrees, Abdulrahman Almehery, Manal AAssiri, Abdulrahman Alnemri, Amira R. Al Darwish, Badi Baqawi, Ahmad Aboshaiqah, Layal Hneiny, Rana H. Almaghrabi, Ahmed M. El-Malky, Nawaf M. Al-Dajani

**Affiliations:** ^1^Pediatrics Department, King Khalid University Hospital, Riyadh, Saudi Arabia; ^2^Clinical Practice Guidelines and Quality Research Unit, Quality Management Department, King Saud University Medical City, Riyadh, Saudi Arabia; ^3^Research Chair for Evidence-Based Health Care and Knowledge Translation, King Saud University, Riyadh, Saudi Arabia; ^4^Alexandria Center for Evidence-Based Clinical Practice Guidelines, Alexandria University, Alexandria, Egypt; ^5^Adaptation Working Group, Guidelines International Network, Perth, Scotland; ^6^Pediatrics Department, College of Medicine, King Saud University, Riyadh, Saudi Arabia; ^7^Neonatal Intensive Care Unit, King Saud University Medical City, Riyadh, Saudi Arabia; ^8^Saudi Neonatology Society (SNS), Riyadh, Saudi Arabia; ^9^Neonatology Department, Ministry of Health, Assir, Saudi Arabia; ^10^Neonatology Department, King Abdulaziz Hospital, Ministry of Health, Jeddah, Saudi Arabia; ^11^Clinical Pharmacy Department, Pharmacy Services, Second Health Cluster in Central Region, Riyadh, Saudi Arabia; ^12^Pharmacy Department, King Fahad Medical City, Ministry of Health, Riyadh, Saudi Arabia; ^13^Obstetrics and Gynecology Department, King Fahad Medical City, Ministry of Health, Riyadh, Saudi Arabia; ^14^College of Nursing, King Saud University, Riyadh, Saudi Arabia; ^15^Saab Medical Library, University Libraries, American University of Beirut, Beirut, Lebanon; ^16^Wegner Health Sciences Library, University of South Dakota, Sioux Falls, SD, United States; ^17^Department of Pediatrics, Prince Sultan Military Medical City, Riyadh, Saudi Arabia; ^18^Morbidity and Mortality Unit, King Saud University Medical City, King Saud University, Riyadh, Saudi Arabia; ^19^Public Health and Community Medicine Department, Theodor Bilharz Research Institute (TBRI), Academy of Scientific Research, Cairo, Egypt; ^20^Neonatal Intensive Care Unit, Infectious Diseases Unit, Pediatrics Department, King Abdulaziz University Hospital, Jeddah, Saudi Arabia

**Keywords:** neonatal sepsis, pediatrics, clinical practice guidelines, systematic review, AGREE II instrument, quality assessment

## Abstract

**Background and objective:**

Neonatal sepsis (NS) continues to be a critical healthcare priority for the coming decades worldwide. The aim of this study was to critically appraise the quality of recent clinical practice guidelines (CPGs) for neonatal sepsis and to summarize and compare their recommendations.

**Methods:**

This study involves a systematic review of CPGs. We identified clinical questions and eligibility criteria and searched and screened for CPGs using bibliographic and CPG databases and professional societies. Each included CPG was assessed by four independent appraisers using the Appraisal of Guidelines for REsearch & Evaluation II (AGREE II) instrument. We summarized the recommendations in a comparison practical table. The systematic review was drafted according to the Preferred Reporting Items for Systematic reviews and Meta-Analyses (PRISMA) statement. Its protocol was registered in the PROSPERO International Prospective Register of Systematic Reviews (ID: CRD42021258732).

**Results:**

Our search retrieved 4,432 citations; of which five CPGs were eligible and appraised: American Academy of Pediatrics (AAP 2018) (35 and 34 weeks); Canadian Pediatric Society (CPS 2017); National Institute for Health and Care Excellence (NICE 2021); and Queensland Maternity and Neonatal Services (QH 2020). Among these, the overall assessment of two evidence-based CPGs scored > 70% (NICE and QH), which was consistent with their higher scores in the six domains of the AGREE II instrument. In domain 3 (rigor of development), NICE and QH scored 99 and 60%, respectively. In domain 5 (applicability), they scored 96 and 74%, respectively, and in domain 6 (editorial independence), they scored 90 and 71%, respectively.

**Conclusion:**

The methodological quality of the NICE CPG was superior followed by the QH CPG with relevant recommendations for use in practice.

**Systematic review registration:**

https://www.crd.york.ac.uk/prospero/display_record.php?ID=CRD42021258732, PROSPERO (CRD42021258732).

## Introduction

Neonatal sepsis (NS) continues to pose significant morbidity and mortality despite the continued advancement in neonatal care (1, 2). Neonatal sepsis is classified into early- and late-onset depending on the timing of infection in days after birth (3). Another classification includes hospital-acquired vs. community-acquired (4, 5).

The global incidence of NS varies, with a population-level estimate of 2,202 per 100,000 live births, with mortality rates ranging from 11 to 19% in high- and middle-income countries (6) and 2.9 to 24 per 1,000 live births in low-income countries (7). Advancement in obstetrical care and universal screening for Group B Streptococcus (GBS) to stratify risk for NS has helped reduce the incidence of sepsis even further (8). Despite the reduction in NS in many countries, it still possesses a serious threat to neonates (9). Neonatal bacterial infection affecting neonates admitted to the neonatal intensive care unit (NICU) further complicates their course in the hospital and increases the risk of morbidity and mortality (10).

Identifying infants at risk for sepsis is crucial to reducing the complications of neonatal sepsis (11). Many organizations have developed clinical practice guidelines (CPGs), protocols, and policies to try to minimize neonatal sepsis (12). Due to the lack of high-quality studies in the management of NS, most of the published CPGs are based on expert opinions and do not really provide clear guidance for the physician taking care of vulnerable neonates (13). The developed CPGs concentrate mostly on early NS risk assessment and provide no guidance to late-onset sepsis including the AAP and CPS CPGs (14).

At present, there is no unified national CPG in Saudi Arabia for the management of sepsis (15). Furthermore, GBS screening for pregnant women is not a standard of care in Saudi Arabia yet (16). A recent cohort study identified the risk for early NS in Saudi Arabia to be 0.5/1,000 live births (17). A unified management plan of at-risk neonates can help further reduce the risk of complications posed by having an early neonatal infection (18). CPGs have been recognized with their potential to improve clinical practice and patient outcomes (19).

In 2021, the Saudi Neonatology Society (SNS) launched a number of projects to adapt national evidence-based CPGs for the management of high-priority health topics in neonatal healthcare, with the goal of providing evidence-based guidance and recommendations to neonatologists and pediatricians across the country. The “KSU-Modified-ADAPTE” as a formal CPG adaptation methodology consisting of three phases, namely, setup, adaptation, and finalization, has guided these projects (20–23).

The Appraisal of Guidelines for REsearch and Evaluation (AGREE II) instrument is the gold standard for assessing the quality of CPGs. AGREE II is a CPG appraisal tool that has been cited and endorsed by a number of healthcare organizations (24–26). AGREE II identifies components that CPGs should address in order to improve their quality and dependability and achieve positive patient outcomes (24–26).

Because systematic reviews of CPGs using AGREE II is a critical step in the CPG adaptation process, we have dedicated this study to report the results of this systematic review and AGREE II assessment of the recently published CPGs for the management (i.e., diagnosis and treatment) of neonatal sepsis (21, 27, 28). This CPG adaptation project was registered in the PREPARE (Practice guideline REgistration for trancPAREncy) platform, University of Lanzhou, Lanzhou, China http://www.guidelines-registry.org/ (Registration Number: IPGRP-2021CN383) (29).

Both the PIPOH (P, Population; I, Intervention; P, Professionals; O, Outcomes; and H, Healthcare setting) and PICAR [P: Population, clinical indication(s), and condition(s), I: Intervention(s), C: Comparator(s), Comparison(s), and (key) Content, A: Attributes of eligible CPGs, and R: Recommendation characteristics] models were used to guide the search strategy (21, 27).

## Methods

The protocol for this study was registered in PROSPERO (International Prospective Register of Systematic Reviews) (Protocol ID: CRD42021258732).

Our Guidelines Review Group (GRG) included seven expert consultant neonatologists: one of them with expertise in infectious diseases, one with expertise in systematic reviews, a consultant in Obstetrics and Gynecology, a clinical pharmacist with expertise in neonatal medication management, a specialized nurse with relevant expertise, a medical and healthcare librarian, and a CPG methodologist with a background in pediatrics.

### Data sources and search strategy

The librarian systematically searched MEDLINE, EMBASE, and CINAHL databases for relevant CPGs using the Ovid platform and hand-searched the relevant CPG databases and repositories for eligible CPGs (refer to search strategy in Supplement 1).

Two reviewers (LS and AH) independently screened the titles and abstracts of CPGs and articles that met the inclusion criteria. Three different reviewers checked the screening and full-text review (YSA, JA, and NA). After retrieving and reviewing the full-text articles or full CPG documents, disagreements were resolved through focus group discussions.

### Inclusion and exclusion criteria

The following were the NS CPGs eligibility requirements: (1) evidence-based, with a clear detailed record of the CPG development methodology. (2) English or Arabic language. (3) original source CPGs (*de novo* development). (4) national or international scope, and (5) published by an organization or group authorship and accessible from a CPG database or peer-reviewed journal or relevant professional society website. Each source CPG was only appraised in its most recent version. Both organism-specific and nonspecific were considered in the search. CPGs that were published prior to 2016, were not in English or Arabic, were adapted from other CPGs, were presented as consensus or expert-based statements, or had a single author were excluded.

### AGREE II instrument training workshop

The CPG methodologist led a capacity-building workshop for the GRG, which included hands-on sessions on evidence-based CPG standards and using the AGREE II instrument. Following that, four reviewers were assigned to score the CPGs that were included. Each CPG was critically appraised independently by each of the four reviewers. All appraisers read the full CPG documents, including any updates with relevant supplementary information or links to online web pages related to CPG methods or implementation tools. The AGREE II appraisers were instructed to record the justifications for their scores in the “Comments” section for each item or question (28).

### AGREE II assessment of NS CPGs

The AGREE II instrument (www.agreetrust.org) has 23 items or questions divided into six domains, namely, scope and purpose, stakeholder involvement, rigor of development, clarity of presentation, applicability, and editorial independence. Using a 7-point Likert scale, each item was scored. The AGREE II evaluation was guided by its online version, “My AGREE PLUS,” which allows for the creation of a CPG appraisal group for each CPG and compiles and calculates the item ratings into domain ratings and comments. Each CPG was critically appraised by four AGREE II raters who were members of the GRG including four clinicians, and one of them was a methodologist (24, 25, 28).

Large discrepancies in the assessors' scores of items or questions (i.e., a difference of more than 3) were resolved through discussion with the GRG. The standardized AGREE domain scores or ratings were calculated automatically by the online My AGREE PLUS. For each AGREE standardized domain score or rating, we agreed on a cutoff point of 70% since the AGREE II does not provide a specific cutoff point to define high- vs. low-quality CPGs, and several cutoff points have been proposed by different CPG appraisal groups. Following the appraisal, greater emphasis was placed on the scores of domains 3 and 5 in order to facilitate the filtration and final evaluation of the reporting quality of the included CPGs. Identical cutoff values have been reported (28, 30–32).

### Data analysis plan

Using the methods recommended by the AGREE II instrument, we calculated standardized scores ranging from 0 to 100% for each AGREE II domain. The key recommendations of the eligible CPGs were summarized in a comparative tabular format. The quality of CPGs was classified based on the rating of domain 3 (rigor of development), with a high-quality CPG receiving a standardized domain rating of more than or equal to 70%, a moderate-quality CPG receiving a domain rating of 40–69%, and a low-quality CPG receiving a domain rating of <40% (28).

### Inter-rater analysis

We used inter-rater reliability tests to determine the degree of agreement between raters (IRR) using a percent agreement inter-rater reliability assessment test for each question in each area of the five appraised CPGs to determine the level of agreement among the four raters. In addition to the percent agreement in the first overall assessment (OA1), we also investigated the consistency of ratings or the capacity for datasets that were gathered as clusters or sorted into clusters using intra-class correlation in the second overall assessment (OA2). Intra-class correlation is a popular IRR approach (ICC). We use this when there are more than two raters. A strong intra-class correlation coefficient (kappa) around 1 suggested that standards from the same set were quite comparable. A low kappa value around 0 indicated that standards from the same set were not similar. We used ANOVA “One-Way Random” on SPSS Statistics, version 21, since we had inconsistent raters/rates. We picked ICCC because of the wide range of numerical data from groups or clusters. This helped us determine the repeatability and how closely peers resembled one another in terms of certain features or attributes. We investigated how well two ordinal scale categories agreed with one another.

We used weighted kappa since the data came from an ordered scale (quadratic weights). The weights are calculated as follows: Cohen's kappa notation is used. Because the difference between the first and second categories was comparable with the difference between the second and third categories, and so on, we chose linear weights. To quantify agreement, the kappa (K) statistic is used (32, 33): When there is total agreement between the categorization systems, *K* = 1 when there is no agreement larger than chance, and *K* is negative when there is agreement poorer than chance. Supplement 3.1. Table illustrates how the *K* value might be interpreted (34).

## Results

### Identification of neonatal sepsis CPGs

A total of 4,432 records were retrieved 469 records were duplicates 3,916 records were excluded by title and abstract review using Rayyan https://www.rayyan.ai/ (35), and 41 records were excluded after full-text review according to the health questions and the eligibility criteria. Only five source original CPGs were found to be eligible for the quality assessment step, namely, Management of Neonates Born at ≥35 0/7 Weeks' Gestation With Suspected or Proven Early-Onset Bacterial Sepsis (AAP 2018) (36), Management of Neonates Born at ≤ 34 6/7 Weeks' Gestation With Suspected or Proven Early-Onset Bacterial Sepsis (AAP2 2018) (37), Canadian Pediatric Society: Management of Term Infants at Increased Risk for Early-Onset Bacterial Sepsis (CPS 2017) (14), National Institute for Health and Care Excellence. Neonatal Infection: Antibiotics for Prevention and Treatment (NICE 2021) (38), and Queensland Health: Early Onset Group B Streptococcal Disease (QH 2020) (39). Two reviewers conducted the screening (LS, AH), and two additional reviewers (JA, YSA) resolved any discrepancies by discussions. The PRISMA flowchart was reported in the online Supplementary material (Supplement 2. Figure).

### Key characteristics of neonatal sepsis CPGs

Table 1 highlights the characteristics of all eligible CPGs. The CPG developer organizations were reference, professional organizations in pediatrics, neonatology, or general non-specialized including AAP, CPS, NICE, and QH. All organizations were from high-income countries.

**Table 1:** Characteristics of included neonatal sepsis CPGs.

**Organization, Country**	**Type of the developer organization**	**Scope of the CPG**	**Health system, economic classification**	**CPG Title**	**Year of publication**	Using the GRADE*Method in development	**Number of references**	Number of cited SRs (N), CSR (n), NCSR (*n*)***
1. American Academy of Pediatrics (AAP 35 W)	Professional Society	National	Private Health System, High-income country	Management of Neonates Born at ≥35 0/7 Weeks' Gestation With Suspected or Proven Early-Onset Bacterial Sepsis^8^	2018	No	83	SR (*N* = 2), CSR (*n* = 0), NCSR (*n* = 2)
2. American Academy of Pediatrics (AAP 34 W)	Professional Society	National	Private Health System, High-income country	Management of Neonates Born at ≤ 34 6/7 Weeks' Gestation With Suspected or Proven Early-Onset Bacterial Sepsis^9^	2018	No	67	SR (*N* = 2), CSR (*n* = 0), NCSR (*n* = 2)
3. Canadian Pediatric Society (CPS)	Professional Society	National	National Health Insurance, High-income country	Management of term infants at increased risk for early-onset bacterial sepsis^10^	2017	No	58	SR (*N* = 2), CSR (*n* = 0), NCSR (*n* = 2)
4. National Institute for Health and Care Excellence (NICE)	Independent, executive, public organization set up by the Government to tackle the variation in availability and quality of healthcare in the NHS	National	National Health Service, High-income country	Neonatal infection: antibiotics for prevention and treatment^4^	2021	Yes GRADE, GRADE-CERQual**	9 Evidence Reviews (with a total of 136 references) were conducted for the updated 2021 CPG. 1. 141 in the previous 2012 CPG.	SR (*N* = 12), CSR (*n* = 2), NCSR (*n* = 1 referenced and 9 newly conducted).
5. Queensland Maternity and Newborn Services (QH)	Professional Society	National	National Health Insurance, High-income country	Early onset Group B Streptococcal disease^11^	2020	No	74	SR (*N* = 6), CSR (*n* = 4), NCSR (*n* = 2)

^*^GRADE, Grading of Recommendations, Assessment, Development and Evaluations.

^**^CERQual, Confidence in the Evidence from Reviews of Qualitative research.

^***^SR, Systematic Reviews (with or without Meta-analysis); CSR, Cochrane Systematic Review; NCSR, Non-Cochrane Systematic Review.

### Reporting the quality of NS CPGs

The AGREE II standardized domain ratings are summarized in Table 2.

**Table 2:** AGREE II assessment results and domain scores for the five included neonatal sepsis CPGs.

**Source CPGs/ AGREE II domains scores (%)**	**AAP (34W)**	**AAP (35W)**	**CPS 2017**	**NICE 2021**	**QH 2021**
**Domain 1. Scope and purpose** Items 1–3: Objectives, Health question(s), Population (patients, public, etc.)	60%	53%	61%	99%	67%
**Domain 2. Stakeholder involvement** Items 4–6: Group Membership, Target population preferences and views, Target users	38%	43%	36%	94%	69%
**Domain 3. Rigor of development** Items 7–14: Search methods, Evidence selection criteria, Strengths and limitations of the evidence, Formulation of recommendations, Consideration of benefits and harms, Link between recommendations and evidence, External review, Updating procedure.	15%	9%	26%	99%	60%
**Domain 4. Clarity and presentation** Items 15–17: Specific and unambiguous recommendations, Management options, Identifiable key recommendations	65%	68%	78%	100%	94%
**Domain 5. Applicability** Items 18–21: Facilitators and barriers to application, Implementation advice/ tools, Resource implications, Monitoring/auditing criteria	9%	7%	20%	96%	74%
**Domain 6. Editorial independence** Items 22, 23: Funding body, Competing interests	56%	56%	4%	90%	71%
**Overall assessment 1** (Overall quality)	33%	38%	38%	100%	75%
**Overall assessment 2** (Recommend the CPG for use by four appraisers)	No (4)	No (4)	No (4)	Yes (4)	Yes (3), Yes with modif.(1)

*AGREE II, Appraisal of Guidelines for Research and Evaluation Version II Instrument; AAP, American Academy of Pediatrics; CPG, Clinical Practice Guideline; CPS, Canadian Pediatric Society; NICE, National Institute of Clinical and Health Excellence; QH, Queensland Health*.

#### Domain 1: Scope and purpose

The range of domain 1 was between 65 and 99%. The score of one CPG was > 70% (NICE = 99%). NICE presented its 13 health questions in the online full CPG document and appendices. The overall objective of the NICE CPG was clearly stated.

#### Domain 2: Stakeholder involvement

The range of domain 2 was between 36 and 94%. The score of one CPG was > 70% (NICE = 94%) where the guideline development group was properly reported and included a multidisciplinary team representing all related disciplines to the health topic of neonatal sepsis.

#### Domain 3: Rigor of development

The range of domain 3 was between 9 and 99%. The score of one CPG was > 70% (NICE = 99%). The NICE CPG followed the NICE manual for CPG development that uses the Grading of Recommendations, Assessment, Development and Evaluations (GRADE) method. It takes account of both clinical and economic evidence in the formulation of the recommendations. Full GRADE and GRADE-CERQual Evidence Tables were provided including the assessment of the five modified GRADE domains (i.e., risk of bias, inconsistency, indirectness, imprecision, and publication bias). The QH CPG reported an overall CPG development process. Both CPGs considered benefits and harms during the formulation of their recommendations.

#### Domain 4: Clarity of presentation

The range of domain 4 was between 65 and 100%. The score of three CPGs were > 70% (NICE = 100%, QH = 94%, CPS = 78%). The three CPGs presented a clear summary of their key recommendations.

#### Domain 5: Applicability

The range of domain 5 was between 7 and 96%. Two CPGs scored > 70% (NICE = 96%, QH = 74%). The NICE CPG reported a full package of CPG implementation tools including clinical pathways, quality standards, baseline assessment tool, visual summary versions of the CPG, a link to the online Kaiser Permanente neonatal sepsis calculator, patient health education information, and a shared learning experience from the United Kingdom. Furthermore, the QH CPG provided a variety of implementation tools like flowcharts, an implementation section, quality measures, safety and quality table, education, consumer information, a set of online learning resources, and audit items.

#### Domain 6: Editorial independence

The range of domain 6 was between 4 and 90%. The scores of two CPGs were > 70% in domain 6 (NICE = 90%, QH = 71%). Documenting the funding body and the conflicts of interest were included in both CPGs.

#### Overall assessment

**Figure 1:**
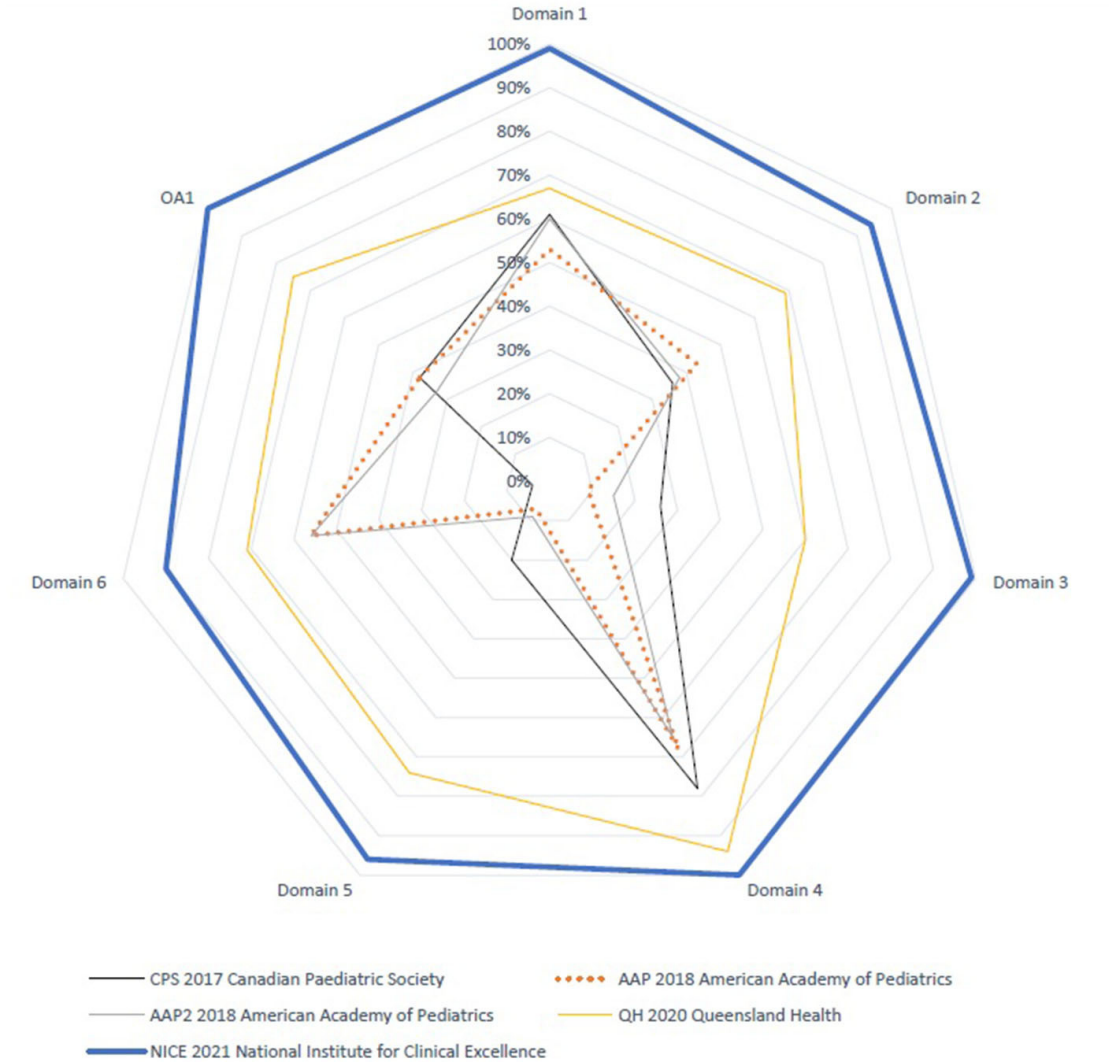
AGREE II domains standardized scores for the five source guidelines.

**Figure 2:**
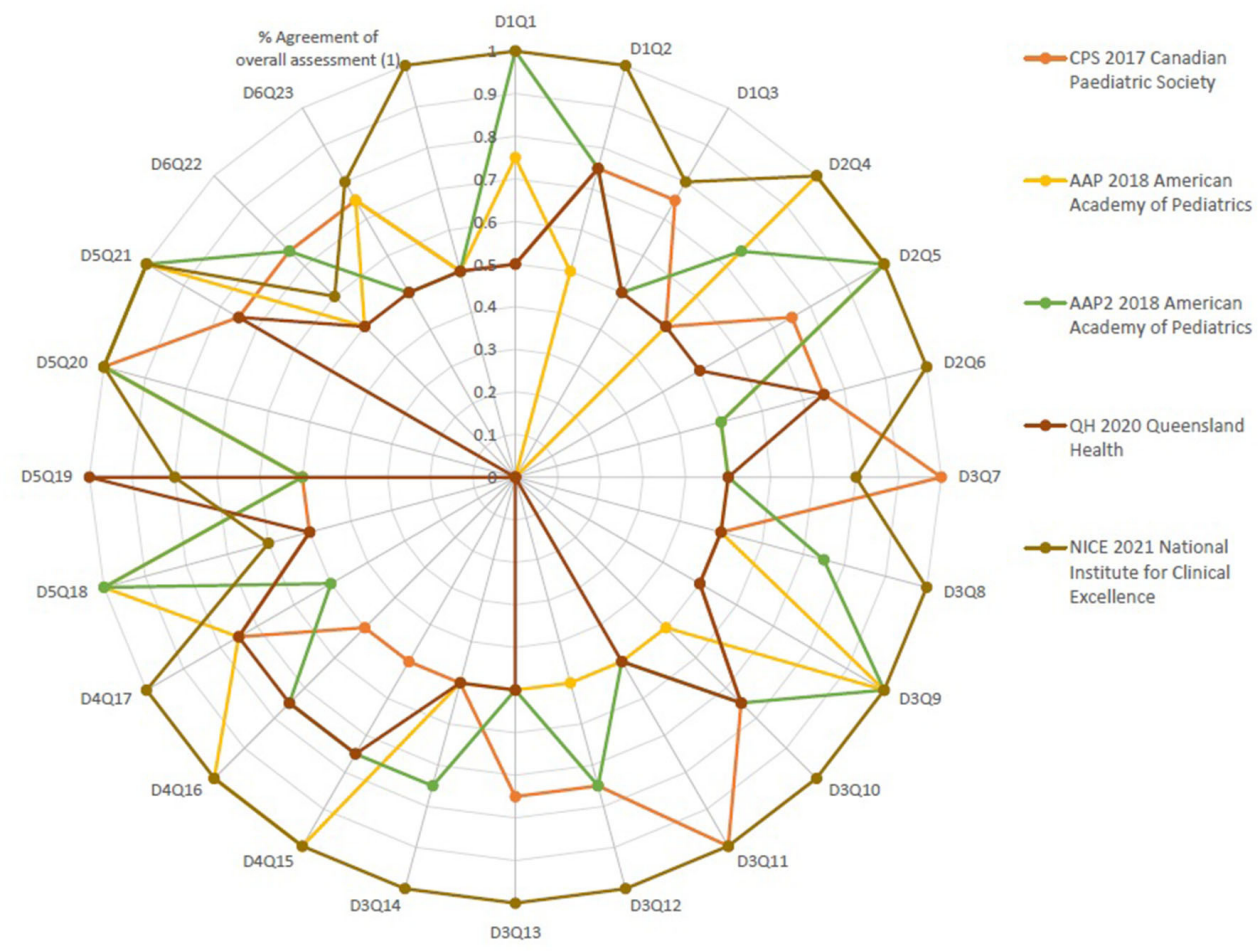
Percent agreement among raters for the five source neonatal sepsis practice guidelines focusing on every question in every domain of the AGREE II Instrument.

The AGREE II standardized domain scores for the first overall assessment ranged from 33 to 100%. Two CPGs scored > 70% (NICE and QH), which was consistent with the high scores in the six AGREE II domains. Figures 1 and 2 display the AGREE II domain scores that were generated. These two radar maps show the appraised CPGs' final percentage scores for each of the six domains and each of the 23 questions in Figures 1 and 2, respectively.

##### Recommending the neonatal sepsis CPGs for use in neonatal practice

The second (overall) assessment (i.e., the recommendation for using the CPG in practice) revealed a consensus between the reviewers on recommending the use of two CPGs (NICE and QH).

### Inter-rater analysis

Table 2 shows the AGREE II group appraisal of the five eligible source CPGs. We calculated the percentage of agreement between raters. The findings of the inter-rater reliability tests revealed a high level of agreement among the four raters for every question in every area in the six domains, as well as the overall assessment's percent agreement. Figure 2, Supplement 3.1. Table and 3.2. Table show that the majority of the kappa scores ranged from 0.50 to 1.00, indicating a good to excellent agreement.

Three assessments, presented in Figure 2, namely, One in AAP 2018 American Academy of Pediatrics and two assessments in QH 2020 Queensland Maternity and Neonatal Clinical Guidelines, found a low level of agreement (*K* = 0.0). No questions in any of the guidelines have a fair degree of agreement. There was very good agreement in 10 questions in CPS 2017 Canadian Pediatric Society, 12 questions in AAP 2018 American Academy of Pediatrics, 9 questions in AAP2 2018 American Academy of Pediatrics guideline, 14 questions in QH 2020 Queensland Maternity and Neonatal Clinical Guidelines, and 2 questions in NICE 2021. There was an excellent degree of agreement in 3 questions in CPS 2017 Canadian Pediatric Society, 8 questions in AAP 2018 American Academy of Pediatrics, 6 questions in AAP2 2018 American Academy of Pediatrics guideline, 1 question in QH 2020 Queensland Maternity and Neonatal Clinical Guidelines, and 18 questions in NICE 2021. Regarding the summation of scores, NICE 2021 showed the highest score of 786 and also 35 was the OA1 score. Overall assessment of NICE 2021 was “Excellent” (weighted kappa score = 1). The intraclass correlation coefficient (kappa value) among raters of the four recommendations for the overall assessment (2)showed that the number of observed agreements is six (61.26% of the observations); 8 agreements are predicted by chance (75% of the observations). Kappa = 0.837; kappa SE = 0.761; 95% confidence interval: weighted kappa = 0.091 for values ranging from 0.214 to 0.617.

## Discussion

Despite the large volume of national and international neonatal CPGs that are continuously published, there exists the challenge of variability of their quality and evidence base. To the best of our knowledge, this review is novel in that it systematically evaluates the quality of recently published CPGs of neonatal sepsis using the AGREE II instrument as a part of a national CPG adaptation initiative (40–43).

Five source CPGs addressing the management of neonatal sepsis were assessed using the AGREE II instrument. This AGREE II assessment highlighted several areas of improvement in the methodological rigor of the included CPGs. Although the assessment of overall guideline quality and the recommendation for use are standard components of AGREE II, it is possible that they are underreported in the documented methodology of the published CPGs.

In this review, the scores of only two CPGs (NICE and QH) were ≥ 60% in domain 3 (rigor of development) that has been identified as the strongest indicator of the quality and evidence base of a CPG more than the other five domains (30). A comparison or a recommendation matrix table was summarized for the five included assessed source CPGs in Table 3.

**Table 3:** Recommendation matrix table for the five eligible source guidelines.

**Title of the assessed source CPGs/ summary of the recommendations**	**National Institute of Care Excellence (NICE) guidelines (20 april 2021) Neonatal infection: antibiotics for prevention and treatment**	**American Academy of Pediatrics (AAP) 2018 CPG (management of neonates born at ≤ 34 6/7 weeks' gestation with suspected or proven early-onset bacterial sepsis)**	**American Academy of Pediatrics (AAP) 2018 CPG (management of neonates born at ≥35 0/7 weeks' gestation with suspected or proven early-onset bacterial sepsis)**	**Canadian Pediatric Society (2017) management of term infants at increased risk for early-onset bacterial sepsis**	**Queensland clinical guideline (2016) early onset group B Streptococcal disease**
**Neonates born at** **≥35 0/7 weeks' gestation**
Risk factors for and clinical indicators of possible early-onset neonatal infection Before birth	Risk factors were provided in the source CPG.	Not mentioned	Acceptable approaches to risk stratification include the following: ° Categorical algorithms in which threshold values for intrapartum risk factors are used ° Multivariate risk assessment based on both intrapartum risk factors and infant examinations. The Neonatal Early-Onset Sepsis Risk Calculator is an example of this approach ° Serial physical examination to detect the presence of clinical signs of illness after birth.	The risk factors associated most frequently with EOS in term infants are summarized and provided in the source CPG.	Risk factors for EOGBSD include: Preterm labor (PTL) at <37+0 weeks (spontaneous or induced) ROM > or equal to 18 h prior to birth Maternal temperature greater than or equal to 38 °C intrapartum or within 24 h of giving birth GBS colonization in the current pregnancy GBS bacteriuria in the current pregnancy Previous baby with EOGBSD
Assessing and managing the risk of early-onset neonatal infection after birth	Clinical assessment Maternal and neonatal history Physical examination of the baby, including an assessment of vital signs.	Not mentioned	Birth centers should consider the development of locally tailored, documented guidelines for EOS risk assessment and clinical management.	Not mentioned	Not mentioned
Management for babies at increased risk of infection	Consider starting antibiotic treatment.	Not mentioned	Not mentioned	In 2007, CPS published recommendations for management of infants at increased risk of EOS	Not mentioned
Investigations before starting antibiotics in babies who may have early-onset infection	Blood culture C-reactive protein Lumbar puncture	Not mentioned	Blood or CSF cultures.	CBC, blood culture and lumber puncture, Infants who have respiratory signs should also have a chest x-ray.	Investigations of sepsis were provided in detailed in the source CPG.
Antibiotics for suspected early-onset infection	IV benzylpenicillin with gentamicin	Not mentioned	Ampicillin and gentamicin. The empirical administration of additional broad-spectrum agents may be indicated in term infants who are critically ill until appropriate couture results are known	Empirical IV Ampicillin and an aminoglycoside	° Benzylpenicillin **Or** amoxicillin/ampicillin °**PLUS** gentamicin
Duration of antibiotic treatment for early-onset neonatal infection Investigations during antibiotic treatment for early-onset neonatal infection	C-reactive protein concentration 18 to 24 h after presentation Lumbar puncture **Decisions 36 h after starting antibiotic treatment** consider stopping the antibiotics at 36 h if: blood culture is negative and the initial clinical suspicion of infection was not strong and the baby's clinical condition is reassuring, with no clinical indicators of possible infection and the levels and trends of C-reactive protein are reassuring. **Treatment duration for early-onset neonatal infection without meningitis**	Not mentioned	When blood cultures are sterile, antibiotic therapy should be discontinued by 36 to 48 h of incubation unless there is clear evidence of site-specific infection.	A CBC done after 4 h of age may be helpful, WBC <5 × 10^9^/L and ANC <1.5 × 10^9^/L have the highest positive predictive value.	Varies depending on results of cultures and clinical course, discuss with a pediatrician or infectious diseases physician If GBS sepsis is proven or suspected, then continue antibiotics for 7–10 days or longer as indicated If blood cultures are negative, white count is normal, symptoms resolve and baby is known to be well then discontinue antibiotics after 36–48 h
	antibiotic for 7 days for babies with a positive blood culture, and for babies with a negative blood culture if sepsis has been strongly suspected. Consider continuing antibiotic treatment for > 7 days if: ∙ the baby has not yet fully recovered or ∙ this is advisable because of the pathogen identified on blood culture).				
**Neonates born at** **≤34 6/7 weeks' gestation**
Risk factors for and clinical indicators of possible early-onset neonatal infection Before birth	Risk factors were provided in the source CPG	Infants born at ≤ 34 6/7 weeks' gestation can be categorized by level of risk for EOS by the circumstances of their preterm birth. Infants born preterm by cesarean delivery because of maternal non-infectious illness or placental insufficiency in the absence of labor, attempts to induce labor, or ROM before delivery are at a relatively low risk for EOS. Depending on the clinical condition of the neonate, physicians should consider the risk/benefit balance of an EOS evaluation and empirical antibiotic therapy	Not mentioned	Not mentioned	AS above
		° Infants born preterm because of maternal cervical incompetence, preterm labor, PROM, clinical concern for IAI, or acute onset of unexplained non-reassuring fetal status are at the highest risk for EOS. ° Such neonates should undergo EOS evaluation with blood c/sand empirical antibiotic treatment. Obstetric and neonatal care providers should communicate and document the circumstances of preterm birth to facilitate EOS risk assessment among preterm infants			
Assessing and managing the risk of early-onset neonatal infection after birth	As above	Clinical centers should consider the development of locally appropriate written guidelines for preterm EOS risk assessment and clinical management. ° After guidelines are implemented, ongoing surveillance, designed to identify low-frequency adverse events and affirm efficacy, is recommended.	Not mentioned	Not mentioned	
Management for babies at increased risk of infection	consider starting antibiotic treatment.		Not mentioned	Not mentioned	
Investigations before starting antibiotics in babies who may have early-onset infection	As above	The diagnosis of EOS is made by a blood or CSF culture. EOS cannot be diagnosed by laboratory tests alone, Such as CBC count or CRP	Not mentioned	Not mentioned	Investigation of sepsis was provided in details in the source CPG.
Antibiotics for suspected early-onset infection	IV benzylpenicillin with gentamicin as the first-choice antibiotic regimen for empirical treatment of suspected early-onset infection	Ampicillin and gentamicin. Empirical administration of additional broad-spectrum antibiotics may be indicated in preterm infants who are severely ill and at a high risk for EOS, particularly after prolonged antepartum maternal antibiotic treatment.	Not mentioned	Not mentioned	Benzylpenicillin Or amoxicillin/ampicillin PLUS gentamicin
Duration of antibiotic treatment for early-onset neonatal infection Investigations during antibiotic treatment for early-onset neonatal infection	As above	When blood cultures are sterile, antibiotic therapy should be discontinued by 36 to 48 h of incubation, unless there is clear evidence of site-specific infection. Persistent cardiorespiratory instability is common among preterm infants with VLBW and is not alone an indication for prolonged empirical antibiotic administration. Laboratory test abnormalities alone rarely justify prolonged empirical antibiotic administration, particularly among preterm infants at a lower risk for EOS.	Not mentioned	Not mentioned	If GBS sepsis is proven or suspected, then continue antibiotics for 7–10 days or longer as indicated If blood cultures are negative, white count is normal, symptoms resolve and baby is known to be well then discontinue antibiotics after 36–48 h
**Late-Onset Sepsis**
Risk factors for and clinical indicators of possible late-onset neonatal infection /hospital acquired infection	When assessing or reviewing a baby: Check for, the possible clinical indicators of late-onset neonatal infection (Indicators are provided) take into account that prematurity, mechanical ventilation, history of surgery and presence of a central catheter are associated with greater risk of late-onset neonatal infection.	Not mentioned			LOD more common in babies with low birth weight and in the early preterm
Timing of antibiotics for late-onset neonatal infection	If a baby needs antibiotic treatment, give this as soon as possible and always within 1 h of the decision to treat.	Not mentioned			
Investigations before starting antibiotics in babies who may have late-onset infection	Blood culture Baseline C-reactive protein Lumbar puncture Do not routinely perform urine microscopy or culture as part of the investigations for late-onset neonatal infection for babies in neonatal units.	Not mentioned			
Antibiotics for late-onset neonatal infection Choice of antibiotics	Combination of narrow-spectrum antibiotics (such as IV flucloxacillin plus gentamicin) as first-line treatment if necrotising enterocolitis is suspected, also include an antibiotic that is active against anaerobic bacteria (such as metronidazole).	Not mentioned			
Duration of antibiotic treatment for late-onset neonatal infection Investigations during antibiotic treatment for late-onset neonatal infection	As above	Not mentioned			
Treatment duration for late-onset neonatal infection without meningitis	As above	Not mentioned			
Antifungals to prevent fungal infection during antibiotic treatment for late-onset neonatal infection	Give prophylactic oral nystatin to babies treated with antibiotics for suspected late-onset neonatal bacterial infection if they: have a birthweight of up to 1,500 g or were born at <30 weeks' gestation. If oral administration of nystatin is not possible, give intravenous fluconazole. In April 2021, this was an off-label use of fluconazole	Not mentioned			
Avoiding routine use of antibiotics in babies	Do not routinely give antibiotic treatment to babies without risk factors for infection or clinical indicators or laboratory evidence of possible infection.	Not mentioned			

The Burden of Antibiotic Resistance in Neonates from Developing Societies (BARNARDS) Study conducted an international prospective observational cohort study across 12 clinical sites that highlighted the burden and high mortality of neonatal sepsis among facility-born neonates in low-income and middle-income countries (44). Based on data from the Global Burden of Disease Study 2019, similar results were obtained (45).

Our study is the first systematic critical appraisal of CPGs with diagnostic and therapeutic recommendations for newborns with sepsis that we are aware of. Strengths of our study included using a comprehensive PRISMA-compliant systematic review methodology to identify potentially relevant CPGs and performed quality assessment using the AGREE II instrument by a multidisciplinary expert team of neonatology clinicians and methodologists.

Nevertheless, some limitations were identified in our work. Earlier disadvantages of the AGREE II instrument have been addressed in the “AGREE-REX” (Recommendation EXcellence) tool, which addresses the clinical credibility of the CPG recommendations (46, 47). Language limitation (i.e., searching only English or Arabic language CPGs) may have resulted in the exclusion of relevant neonatal sepsis CPGs that were intended for use in non-English-speaking and non-Arabic contexts.

### Implications for practice: Guidance for clinical guideline uptake

The findings of this review can be further used to inform and support any relevant CPG development or adaptation project for neonatal sepsis.

We recommend including the AGREE II criteria in the capacity building of clinicians to guide their decisions in selecting high-quality and evidence-based CPGs for use in their daily practice through evidence scouting and searching for similar published AGREE II assessments of CPGs in their needed neonatology health topic.

Furthermore, we recommend building a recommendation map (or RecMap) for high-priority health topics or, if possible, for all published neonatology CPGs similar to RecMap initiative for COVID-19 CPGs and for tuberculosis CPGs to increase the accessibility of pre-appraised and living specialized CPGs by the professionals, parents, carers, and the public (48, 49).

### Implications for future CPG research

We recommend conducting research projects to further explore the impact of high or low quality of the NS CPGs on their implementability and implementation including facilitators and barriers in different healthcare contexts, especially in low-resource settings.

## Conclusion

The methodological quality of the NICE and QH CPGs was superior, followed by CPS and AAP CPGs. Recommendations included identification of risk factors, initial assessment, investigations, antibiotic therapy, and treatment of the two main types of neonatal sepsis (i.e., early onset and late onset).

## Data availability statement

The original contributions presented in the study are included in the article/Supplementary material, further inquiries can be directed to the corresponding author.

## Author contributions

YA and JA conceptualized and designed the study. LH, YA, LS, AH, JA, and NA contributed to the search, screening, review, and critical appraisal of guidelines. YA, LS, and AE wrote the first draft of the manuscript. YA and AE analyzed and interpreted the data. YA and JA supervised the procedures in the study and reviewed the drafts and final version of this manuscript. All authors have made substantial contributions and provided final approval for the conception, drafting, and final version of this manuscript. All authors have read and approved the final version of the manuscript.

## Funding

This study was funded by the Saudi Neonatology Society (SNS).

## Conflict of interest

The authors declare that the research was conducted in the absence of any commercial or financial relationships that could be construed as a potential conflict of interest.

## Publisher's note

All claims expressed in this article are solely those of the authors and do not necessarily represent those of their affiliated organizations, or those of the publisher, the editors and the reviewers. Any product that may be evaluated in this article, or claim that may be made by its manufacturer, is not guaranteed or endorsed by the publisher.

## Abbreviations

AGREE II: Appraisal of Guidelines for REsearch and Evaluation (Version 2)
AAP: American Academy of Pediatrics
CPG: Clinical Practice Guideline
CPS: Canadian Pediatric Society
GRADE: Grading of Recommendations; Assessment; Development and Evaluations
GRADE-CERQual: Confidence in the Evidence from Reviews of Qualitative research
NICE: National Institute for Health and Care Excellence
NICU: Neonatal Intensive Care Unit
PRISMA: Preferred Reporting Items for Systematic reviews and Meta-Analyses Statement
PROSPERO: International Database of Prospectively Registered Systematic Reviews with a Health Related Outcome
RecMap: Recommendation Map
QH: Queensland Health (maternity and newborn).
