# Supplementary material for: Quality assessment of clinical practice guidelines for neonatal sepsis using the Appraisal of Guidelines for Research and Evaluation (AGREE) II Instrument: A systematic review of neonatal guidelines
Source: Front Pediatr. 2022 Aug 16;10:891572. doi: 10.3389/fped.2022.891572 (PMC9424847; doi:10.3389/fped.2022.891572)
Supplement: Supplementary file 1 [file Data_Sheet_1.docx]

Supplementary Material

# Supplementary Data

## *Supplement 1. Search strategy*

**Keywords:** neonatal sepsis, neonatal infection, early-onset sepsis, late-onset sepsis, healthcare-associated sepsis, sepsis, bacteremia, meningitis, urinary tract infection, newborn pneumonia, osteomyelitis, cellulitis, guidelines, guidance, recommendations.

**List of the websites and databases we searched**

**CPG databases and libraries:**

1. Guidelines International Network (GIN) International Guidelines Library.

- <https://g-i-n.net/international-guidelines-library/>

1. ECRI Guidelines Trust (USA). <https://guidelines.ecri.org/>
2. National Institute of Clinical and Health Excellence (NICE) UK. <http://www.nice.org.uk/guidance/>
3. Scottish Intercollegiate Guidelines Network (SIGN) UK. <http://www.sign.ac.uk/guidelines/>
4. EBSCO DynaMed (USA) <https://www.dynamed.com/> (subscription required)

**Bibliographic databases:**

1. PubMed/ MEDLINE <https://pubmed.ncbi.nlm.nih.gov/>
2. Embase <https://www.embase.com/landing?status=grey> (subscription required)
3. CINAHL <https://www.ebsco.com/products/research-databases/cinahl-complete>

**Specialized professional societies:**

1. American Academy of Pediatrics (AAP) <https://www.aap.org/>
2. Canadian Paediatric Society (CPS) <https://www.cps.ca/>
3. British Association of Perinatal Medicine (BAPM) <https://www.bapm.org/>
4. Royal College of Paediatrics and Child Health (RCPCH) <https://www.rcpch.ac.uk/>
5. Saudi Neonatology Society (SNS) <https://sns.med.sa/>

### Search strategy for CINAHL

**Limiters** - Published Date: 20110101-20211231---------------------------------------------**Results: 1,623**

| # | Query | Limiters/Expanders | Last Run Via | Results |
| --- | --- | --- | --- | --- |
| S29 | S3 AND S6 AND S28 | Expanders - Apply equivalent subjects Search modes - Boolean/Phrase | Interface - EBSCOhost Research Databases Search Screen - Advanced Search Database - CINAHL Complete | 2,544 |
| S28 | S7 OR S8 OR S9 OR S10 OR S11 OR S12 OR S13 OR S14 OR S15 OR S16 OR S17 OR S18 OR S19 OR S20 OR S21 OR S22 OR S23 OR S24 OR S25 OR S26 OR S27 | Expanders - Apply equivalent subjects Search modes - Boolean/Phrase | Interface - EBSCOhost Research Databases Search Screen - Advanced Search Database - CINAHL Complete | 329,747 |
| S27 | TI ventilator-associated-PNEUMONIA OR AB ventilator-associated-PNEUMONIA OR MW ventilator-associated-PNEUMONIA | Expanders - Apply equivalent subjects Search modes - Boolean/Phrase | Interface - EBSCOhost Research Databases Search Screen - Advanced Search Database - CINAHL Complete | 3,107 |
| S26 | (MH "Pneumonia, Ventilator-Associated") | Expanders - Apply equivalent subjects Search modes - Boolean/Phrase | Interface - EBSCOhost Research Databases Search Screen - Advanced Search Database - CINAHL Complete | 3,522 |
| S25 | TI ( Cellulit* or phlegm#n ) OR AB ( Cellulit* or phlegm#n ) OR MW ( Cellulit* or phlegm#n ) | Expanders - Apply equivalent subjects Search modes - Boolean/Phrase | Interface - EBSCOhost Research Databases Search Screen - Advanced Search Database - CINAHL Complete | 3,396 |
| S24 | (MH "Cellulitis") | Expanders - Apply equivalent subjects Search modes - Boolean/Phrase | Interface - EBSCOhost Research Databases Search Screen - Advanced Search Database - CINAHL Complete | 1,866 |
| S23 | TI ( Mening* or endocardit* ) OR AB ( Mening* or endocardit* ) OR MW ( Mening* or endocardit* ) | Expanders - Apply equivalent subjects Search modes - Boolean/Phrase | Interface - EBSCOhost Research Databases Search Screen - Advanced Search Database - CINAHL Complete | 25,851 |
| S22 | (MH "Endocarditis") OR (MH "Endocarditis, Bacterial") | Expanders - Apply equivalent subjects Search modes - Boolean/Phrase | Interface - EBSCOhost Research Databases Search Screen - Advanced Search Database - CINAHL Complete | 4,553 |
| S21 | (MH "Meningitis+") | Expanders - Apply equivalent subjects Search modes - Boolean/Phrase | Interface - EBSCOhost Research Databases Search Screen - Advanced Search Database - CINAHL Complete | 7,462 |
| S20 | TI ( ((antifungal or anti-fungal or fungicides) N2 (therapeutic# or agent# or antibiotic#) ) OR AB ( ((antifungal or anti-fungal or fungicides) N2 (therapeutic# or agent# or antibiotic#) ) OR MW ( ((antifungal or anti-fungal or fungicides) N2 (therapeutic# or agent# or antibiotic#) ) | Expanders - Apply equivalent subjects Search modes - Boolean/Phrase | Interface - EBSCOhost Research Databases Search Screen - Advanced Search Database - CINAHL Complete | 7,292 |
| S19 | (MH "Antifungal Agents") | Expanders - Apply equivalent subjects Search modes - Boolean/Phrase | Interface - EBSCOhost Research Databases Search Screen - Advanced Search Database - CINAHL Complete | 6,522 |
| S18 | TI Mycos#s OR AB Mycos#s OR MW Mycos#s | Expanders - Apply equivalent subjects Search modes - Boolean/Phrase | Interface - EBSCOhost Research Databases Search Screen - Advanced Search Database - CINAHL Complete | 5,836 |
| S17 | (MH "Mycoses") | Expanders - Apply equivalent subjects Search modes - Boolean/Phrase | Interface - EBSCOhost Research Databases Search Screen - Advanced Search Database - CINAHL Complete | 4,708 |
| S16 | TI ( (Endotoxemia# or Fung* or Candidemia# or (endotoxin N0 shock#) or ((septic or toxic) N0 shock#) or (blood N0 poison*) or (bloodstream N0 infection#) or py#emia# or pyohemia# or seps#s or septic* or Bacter* or uroseps#s) ) OR AB ( (Endotoxemia# or Fung* or Candidemia# or (endotoxin N0 shock#) or ((septic or toxic) N0 shock#) or (blood N0 poison*) or (bloodstream N0 infection#) or py#emia# or pyohemia# or seps#s or septic* or Bacter* or uroseps#s) ) OR MW ( (Endotoxemia# or Fung* or Candidemia# or (endotoxin N0 shock#) or ((septic or toxic) N0 shock#) or (blood N0 poison*) or (bloodstream N0 infection#) or py#emia# or pyohemia# or seps#s or septic* or Bacter* or uroseps#s) ) | Expanders - Apply equivalent subjects Search modes - Boolean/Phrase | Interface - EBSCOhost Research Databases Search Screen - Advanced Search Database - CINAHL Complete | 147,707 |
| S15 | (MH "Sepsis+") | Expanders - Apply equivalent subjects Search modes - Boolean/Phrase | Interface - EBSCOhost Research Databases Search Screen - Advanced Search Database - CINAHL Complete | 29,327 |
| S14 | (MH "Urinary Tract Infections") | Expanders - Apply equivalent subjects Search modes - Boolean/Phrase | Interface - EBSCOhost Research Databases Search Screen - Advanced Search Database - CINAHL Complete | 10,420 |
| S13 | TI (urinary N2 infect*) OR AB (urinary N2 infect*) OR MW (urinary N2 infect*) | Expanders - Apply equivalent subjects Search modes - Boolean/Phrase | Interface - EBSCOhost Research Databases Search Screen - Advanced Search Database - CINAHL Complete | 16,054 |
| S12 | (MH "Abscess+") | Expanders - Apply equivalent subjects Search modes - Boolean/Phrase | Interface - EBSCOhost Research Databases Search Screen - Advanced Search Database - CINAHL Complete | 7,633 |
| S11 | TI ( (Abscess* OR (Toxoplasmos#s N2 Cerebr*)) ) OR AB ( (Abscess* OR (Toxoplasmos#s N2 Cerebr*)) ) OR MW ( (Abscess* OR (Toxoplasmos#s N2 Cerebr*)) ) | Expanders - Apply equivalent subjects Search modes - Boolean/Phrase | Interface - EBSCOhost Research Databases Search Screen - Advanced Search Database - CINAHL Complete | 13,593 |
| S10 | TI osteomyelit* OR AB osteomyelit* OR MW osteomyelit* | Expanders - Apply equivalent subjects Search modes - Boolean/Phrase | Interface - EBSCOhost Research Databases Search Screen - Advanced Search Database - CINAHL Complete | 5,593 |
| S9 | (MH "Viruses+") | Expanders - Apply equivalent subjects Search modes - Boolean/Phrase | Interface - EBSCOhost Research Databases Search Screen - Advanced Search Database - CINAHL Complete | 65,981 |
| S8 | TI ( virus* OR viral ) OR AB ( virus* OR viral ) OR MW ( virus* OR viral ) | Expanders - Apply equivalent subjects Search modes - Boolean/Phrase | Interface - EBSCOhost Research Databases Search Screen - Advanced Search Database - CINAHL Complete | 118,168 |
| S7 | TI ( ((neonat* OR "blood stream") N2 infection#) ) OR AB ( ((neonat* OR "blood stream") N2 infection#) ) OR MW ( ((neonat* OR "blood stream") N2 infection#) ) | Expanders - Apply equivalent subjects Search modes - Boolean/Phrase | Interface - EBSCOhost Research Databases Search Screen - Advanced Search Database - CINAHL Complete | 2,393 |
| S6 | S4 OR S5 | Expanders - Apply equivalent subjects Search modes - Boolean/Phrase | Interface - EBSCOhost Research Databases Search Screen - Advanced Search Database - CINAHL Complete | 508,982 |
| S5 | TI ( (baby OR babies OR nicu OR neaonat* OR perinatal OR preemi* OR permaturity OR prematurity OR premi OR premie# OR preterm OR pre-term OR newborn* OR (low W0 birth W0 weight) OR infan* OR newborn# OR new-born# OR (full W0 term) OR LBW OR VLBW) OR AB ( (baby OR babies OR nicu OR neaonat* OR perinatal OR preemi* OR permaturity OR prematurity OR premi OR premie# OR preterm OR pre-term OR newborn* OR (low W0 birth W0 weight) OR infan* OR newborn# OR new-born# OR (full W0 term) OR LBW OR VLBW) OR MW ( (baby OR babies OR nicu OR neaonat* OR perinatal OR preemi* OR permaturity OR prematurity OR premi OR premie# OR preterm OR pre-term OR newborn* OR (low W0 birth W0 weight) OR infan* OR newborn# OR new-born# OR (full W0 term) OR LBW OR VLBW) | Expanders - Apply equivalent subjects Search modes - Boolean/Phrase | Interface - EBSCOhost Research Databases Search Screen - Advanced Search Database - CINAHL Complete | 508,982 |
| S4 | (MH "Infant") OR (MH "Infant, Newborn+") | Expanders - Apply equivalent subjects Search modes - Boolean/Phrase | Interface - EBSCOhost Research Databases Search Screen - Advanced Search Database - CINAHL Complete | Display |
| S3 | S1 OR S2 | Expanders - Apply equivalent subjects Search modes - Boolean/Phrase | Interface - EBSCOhost Research Databases Search Screen - Advanced Search Database - CINAHL Complete | Display |
| S2 | ( TI ( (algorithm* N1 (screening OR examination OR test OR tested OR testing OR assessment* OR diagnosis OR diagnoses OR diagnosed OR diagnosing)) ) OR AB ( (algorithm* N1 (screening OR examination OR test OR tested OR testing OR assessment* OR diagnosis OR diagnoses OR diagnosed OR diagnosing)) ) OR MW ( (algorithm* N1 (screening OR examination OR test OR tested OR testing OR assessment* OR diagnosis OR diagnoses OR diagnosed OR diagnosing)) ) ) OR ( TI ( (algorithm* N1 (pharmacotherap* OR chemotherap* OR chemotreatment* OR therap* OR treatment* OR intervention*) ) OR AB ( (algorithm* N1 (pharmacotherap* OR chemotherap* OR chemotreatment* OR therap* OR treatment* OR intervention*) ) OR MW ( (algorithm* N1 (pharmacotherap* OR chemotherap* OR chemotreatment* OR therap* OR treatment* OR intervention*) ) ) | Expanders - Apply equivalent subjects Search modes - Boolean/Phrase | Interface - EBSCOhost Research Databases Search Screen - Advanced Search Database - CINAHL Complete | Display |
| S1 | ( (MH "Consensus") OR (MH "Consensual Validity") ) OR (MH "Critical Path") OR (MH "Practice Guidelines") OR ( PT guideline OR consensus ) OR ( TI ( ((position or policy) W0 statement*) OR (practice W0 parameter*) OR (best W0 practice*) ) OR AB ( ((position or policy) W0 statement*) OR (practice W0 parameter*) OR (best W0 practice*) ) OR MW ( ((position or policy) W0 statement*) OR (practice W0 parameter*) OR (best W0 practice*) ) ) OR ( TI ( standards OR guideline OR guidelines ) OR MW ( standards OR guideline OR guidelines ) ) OR ( AB ((practice OR treatment* OR clinical) W0 guideline*) ) OR ( TI CPG OR CPGs ) OR ( TI consensus* OR AB consensus* OR MW consensus* ) OR ( TI ( ((critical OR clinical OR practice) N1 (path OR paths OR pathway OR pathways OR protocol*)) ) OR AB ( ((critical OR clinical OR practice) N1 (path OR paths OR pathway OR pathways OR protocol*)) ) OR MW ( ((critical OR clinical OR practice) N1 (path OR paths OR pathway OR pathways OR protocol*)) ) ) OR ( TI recommendat* OR MW recommendat* ) OR ( TI ( (care N1 (standard OR path OR paths OR pathway OR pathways OR map OR maps OR plan OR plans)) ) OR AB ( (care N1 (standard OR path OR paths OR pathway OR pathways OR map OR maps OR plan OR plans)) ) OR MW ( (care N1 (standard OR path OR paths OR pathway OR pathways OR map OR maps OR plan OR plans)) ) ) | Expanders - Apply equivalent subjects Search modes - Boolean/Phrase | Interface - EBSCOhost Research Databases Search Screen - Advanced Search Database - CINAHL Complete | Display |

Bottom of Form

### Search strategy for MEDLINE

**Results 2,817**

Database: Ovid MEDLINE(R) and Epub Ahead of Print, In-Process, In-Data-Review & Other Non-Indexed Citations and Daily <1946 to April 09, 2021>

Search Strategy:

--------------------------------------------------------------------------------

1 exp sepsis/ (126941)

2 (Endotoxemia? or Fung* or Candidemia? or (endotoxin adj1 shock?) or ((septic or toxic) adj1 shock?) or (blood adj1 poison*) or (bloodstream adj1 infection?) or py?emia? or pyohemia? or seps?s or septic* or Bacter* or uroseps?s).mp. (2002707)

3 Mycoses/ or Mycos?s.mp. (36859)

4 Antifungal Agents/ or Invasive Fungal Infections/ (60218)

5 ((antifungal or anti-fungal or fungicides) adj3 (therapeutic? or agent? or antibiotic?)).mp. (65086)

6 exp Meningitis/ or endocarditis/ or exp endocarditis, bacterial/ (84594)

7 (Mening* or endocardit*).mp. (182096)

8 Cellulitis/ or (Cellulit* or phlegm?n).mp. (14659)

9 PNEUMONIA, VENTILATOR-ASSOCIATED/ (3645)

10 (ventilator-associated adj PNEUMONIA).mp. (5641)

11 Osteomyelit*.mp. (29694)

12 urinary tract infection/ or (urinary adj3 infect*).mp. (65701)

13 exp Abscess/ (56961)

14 (Abscess* or (Toxoplasmos?s adj3 Cerebr*)).mp. (97424)

15 osteomyelit*.mp. (29694)

16 exp viruses/ or (virus* or viral).mp. (1285059)

17 ((neonat* or "blood stream") adj3 infection?).mp. (9620)

18 or/1-17 (3407469)

19 exp clinical pathway/ or exp clinical protocol/ or exp consensus/ or exp consensus development conference/ or exp consensus development conferences as topic/ or critical pathways/ or exp guideline/ or guidelines as topic/ or exp practice guideline/ or practice guidelines as topic/ or health planning guidelines/ or (guideline or practice guideline or consensus development conference or consensus development conference, NIH).pt. or (position statement* or policy statement* or practice parameter* or best practice*).ti,ab,kf,kw. or (standards or guideline or guidelines).ti,kf,kw. or ((practice or treatment* or clinical) adj guideline*).ab. or (CPG or CPGs).ti. or consensus*.ti,kf,kw. or consensus*.ab. /freq=2 or ((critical or clinical or practice) adj2 (path or paths or pathway or pathways or protocol*)).ti,ab,kf,kw. or recommendat*.ti,kf,kw. or (care adj2 (standard or path or paths or pathway or pathways or map or maps or plan or plans)).ti,ab,kf,kw. or (algorithm* adj2 (screening or examination or test or tested or testing or assessment* or diagnosis or diagnoses or diagnosed or diagnosing)).ti,ab,kf,kw. or (algorithm* adj2 (pharmacotherap* or chemotherap* or chemotreatment* or therap* or treatment* or intervention*)).ti,ab,kf,kw. (651864)

20 exp Infant/ (1162139)

21 (baby or babies or nicu or neaonat* or perinatal or preemi* or permaturity or prematurity or premi or premie? or preterm or pre-term or (low adj birth adj weight) or infan* or newborn? or new-born? or (full adj term) or LBW or VLBW).mp. (1548886)

22 20 or 21 (1548886)

23 18 and 19 and 22 (5504)

24 limit 23 to yr="2011 -Current" (2817)

***************************

1.

Impact of the Implementation of a Vancomycin Protocol on Trough Serum Vancomycin Concentrations in a Pediatric Intensive Care Unit.

Rezende RQ, Dias CAG, Ricachinevsky CP, de Lucena Capelari JP

Paediatric Drugs. 2021 Apr 08.

[Journal Article]

UI: 33830468

BACKGROUND: Vancomycin is an antibiotic that is widely used in pediatric intensive care, but the safe and effective use of this drug is challenging.

OBJECTIVE: This study aimed to assess the impact of a vancomycin protocol on trough serum concentrations.

METHODS: We conducted a retrospective quasiexperimental study in patients aged <= 18 years in intensive care who received vancomycin for at least 5 days. Patients were divided into two groups: before and after a protocol implemented in 2017 that suggested an initial vancomycin dose of 60 mg/kg/day, target serum levels of 15-20 mug/mL, and dose adjustments. We compared patient characteristics, target serum level achievement, and vancomycin levels over time.

RESULTS: Each group contained 65 patients; most were male infants with heart disease as the main reason for hospitalization. Only 29.2% of the patients had pretreatment cultures for bacteria identification recorded, with 1.5% identified as methicillin-resistant Staphylococcus aureus. For the first serum levels, 10.8% of patients in the pre-protocol group and 21.5% in the post-protocol group achieved the 15-20 mug/mL target (p = 0.153); during the first 5 days of treatment, this proportion significantly increased from 52.3 to 73.8% (p = 0.018). We observed a difference between the first and fifth levels: 8.9 mug/mL (95% confidence interval [CI] - 3.1 to 21) pre-protocol and 0.4 mug/mL (95% CI - 6.1 to 6.9) post-protocol (p = 0.175).

CONCLUSIONS: Reaching adequate trough vancomycin concentrations in critically ill pediatric patients remains a challenge, and clinical practice protocols allow better dose adjustment and control even when monitoring technologies are unavailable.

Version ID

1

Record Owner

From MEDLINE, a database of the U.S. National Library of Medicine.

Status

Publisher

Author NameID

Rezende, Raissa Queiroz; ORCID: <http://orcid.org/0000-0002-5780-4531>

Authors Full Name

Rezende, Raissa Queiroz, Dias, Cicero Armidio Gomes, Ricachinevsky, Claudia Pires, de Lucena Capelari, Joao Paulo

Institution

Rezende, Raissa Queiroz. Pediatric Intensive Care Unit, Hospital da Crianca Santo Antonio, Irmandade Santa Casa de Misericordia de Porto Alegre, Unidade de Terapia Intensiva Pediatrica, Avenida Independencia, 155, Porto Alegre, RS, CEP 90035-074, Brazil. raissaq@gmail.com. Dias, Cicero Armidio Gomes. Department of Basic Health Sciences, Federal University of Health Sciences of Porto Alegre (UFCSPA), Porto Alegre, Brazil.

Ricachinevsky, Claudia Pires. Pediatric Intensive Care Unit, Hospital da Crianca Santo Antonio, Irmandade Santa Casa de Misericordia de Porto Alegre, Unidade de Terapia Intensiva Pediatrica, Avenida Independencia, 155, Porto Alegre, RS, CEP 90035-074, Brazil.

de Lucena Capelari, Joao Paulo. Hospital da Crianca Santo Antonio, Irmandade Santa Casa de Misericordia de Porto Alegre, Porto Alegre, Brazil.

Year of Publication

2021

Link to the Ovid Full Text or citation:

[Click here for full text options](https://ovidsp.ovid.com/ovidweb.cgi?T=JS&CSC=Y&NEWS=N&PAGE=fulltext&D=medp&AN=33830468)

Link to the External Link Resolver:

[More Full text Options](http://vp9py7xf3h.search.serialssolutions.com/?sid=OVID:medline&id=pmid:33830468&id=doi:10.1007%2Fs40272-021-00445-3&issn=1174-5878&isbn=&volume=&issue=&spage=&pages=&date=2021&title=Paediatric+Drugs&atitle=Impact+of+the+Implementation+of+a+Vancomycin+Protocol+on+Trough+Serum+Vancomycin+Concentrations+in+a+Pediatric+Intensive+Care+Unit.&aulast=Rezende&pid=%3Cauthor%3ERezende+RQ%3BDias+CAG%3BRicachinevsky+CP%3Bde+Lucena+Capelari+JP%3C%2Fauthor%3E%3CAN%3E33830468%3C%2FAN%3E%3CDT%3EJournal+Article%3C%2FDT%3E)

### Search strategy for EMBASE

**Results 3,972**

| No.,Query,Results,Date |  |  |  |  |  |  |  |  |  |  |  |  |  |  |  |  |  |  |
| --- | --- | --- | --- | --- | --- | --- | --- | --- | --- | --- | --- | --- | --- | --- | --- | --- | --- | --- |
| #37,"#36 AND (2011:py OR 2012:py OR 2013:py OR 2014:py OR 2015:py OR 2016:py OR 2017:py OR 2018:py OR 2019:py OR 2020:py OR 2021:py)",3972,10 Apr 2021 | | | | | | | | | | | | |  |  |  |  |  |  |
| #36,"#14 AND #30 AND #35",6275,10 Apr 2021 | | |  |  |  |  |  |  |  |  |  |  |  |  |  |  |  |  |
| #35,"#31 OR #32 OR #33 OR #34",1576975,10 Apr 2021 | | | |  |  |  |  |  |  |  |  |  |  |  |  |  |  |  |
| #34,"'new-born':ti,ab,kw OR 'full-term':ti,ab,kw OR lbw:ti,ab,kw OR vlbw:ti,ab,kw",39019,10 Apr 2021 | | | | | | | |  |  |  |  |  |  |  |  |  |  |  |
| #33,"preterm:ti,ab,kw OR 'pre term':ti,ab,kw OR 'low-birth-weight':ti,ab,kw OR infan*:ti,ab,kw OR newborn$:ti,ab,kw",778393,10 Apr 2021 | | | | | | | | | | |  |  |  |  |  |  |  |  |
| #32,"baby:ti,ab,kw OR babies:ti,ab,kw OR nicu:ti,ab,kw OR neaonat*:ti,ab,kw OR perinatal:ti,ab,kw OR preemi*:ti,ab,kw OR permaturity:ti,ab,kw OR prematurity:ti,ab,kw OR premi:ti,ab,kw OR premie$:ti,ab,kw",248888,10 Apr 2021 | | | | | | | | | | | | | | | | | | |
| #31,"'infant'/exp",1173948,10 Apr 2021 | |  |  |  |  |  |  |  |  |  |  |  |  |  |  |  |  |  |
| #30,"#15 OR #16 OR #17 OR #18 OR #19 OR #20 OR #21 OR #22 OR #23 OR #24 OR #25 OR #26 OR #27 OR #28 OR #29",1027920,10 Apr 2021 | | | | | | | | | | |  |  |  |  |  |  |  |  |
| #29,"(algorithm* NEAR/1 (pharmacotherap* OR chemotherap* OR chemotreatment* OR therap* OR treatment* OR intervention*)):ti,ab,kw",13841,10 Apr 2021 | | | | | | | | | | | | |  |  |  |  |  |  |
| #28,"(algorithm* NEAR/1 (screening OR examination OR test OR tested OR testing OR assessment* OR diagnosis OR diagnoses OR diagnosed OR diagnosing)):ti,ab,kw",5455,10 Apr 2021 | | | | | | | | | | | | | | |  |  |  |  |
| #27,"(care NEAR/1 (standard OR path OR paths OR pathway$ OR map$ OR plan$)):ti,ab,kw",39884,10 Apr 2021 | | | | | | | | |  |  |  |  |  |  |  |  |  |  |
| #26,"recommendat*:ti,kw",57002,10 Apr 2021 | | |  |  |  |  |  |  |  |  |  |  |  |  |  |  |  |  |
| #25,"((critical OR clinical OR practice) NEAR/1 (path OR paths OR pathway OR pathways OR protocol*)):ti,ab,kw",20420,10 Apr 2021 | | | | | | | | | | |  |  |  |  |  |  |  |  |
| #24,"consensus*:ti,ab,kw",236742,10 Apr 2021 | | |  |  |  |  |  |  |  |  |  |  |  |  |  |  |  |  |
| #23,"cpg:ti OR cpgs:ti",7110,10 Apr 2021 | |  |  |  |  |  |  |  |  |  |  |  |  |  |  |  |  |  |
| #22,"((practice OR treatment* OR clinical) NEXT/1 guideline*):ab",65619,10 Apr 2021 | | | | | |  |  |  |  |  |  |  |  |  |  |  |  |  |
| #21,"standards:ti,kw OR guideline:ti,kw OR guidelines:ti,kw",162292,10 Apr 2021 | | | | | |  |  |  |  |  |  |  |  |  |  |  |  |  |
| #20,"'position statement*':ti,ab,kw OR 'policy statement*':ti,ab,kw OR 'practice parameter*':ti,ab,kw OR 'best practice*':ti,ab,kw",53051,10 Apr 2021 | | | | | | | | | | | |  |  |  |  |  |  |  |
| #19,"guideline:it OR consensus:it",0,10 Apr 2021 | | |  |  |  |  |  |  |  |  |  |  |  |  |  |  |  |  |
| #18,"'practice guideline'/exp",590955,10 Apr 2021 | | |  |  |  |  |  |  |  |  |  |  |  |  |  |  |  |  |
| #17,"guideline:it OR 'practice guideline':it OR 'consensus development conference':it OR 'consensus development conference, nih':it",0,10 Apr 2021 | | | | | | | | | | | |  |  |  |  |  |  |  |
| #16,"'guideline'/exp",141,10 Apr 2021 | |  |  |  |  |  |  |  |  |  |  |  |  |  |  |  |  |  |
| #15,"'clinical pathway'/exp OR 'clinical protocol'/exp OR 'consensus'/exp",189771,10 Apr 2021 | | | | | | |  |  |  |  |  |  |  |  |  |  |  |  |
| #14,"#1 OR #2 OR #3 OR #4 OR #5 OR #6 OR #7 OR #8 OR #9 OR #10 OR #11 OR #12 OR #13",2459593,10 Apr 2021 | | | | | | | | |  |  |  |  |  |  |  |  |  |  |
| #13,"((neonat* OR 'blood stream') NEAR/3 infection$):ti,ab,kw",14332,10 Apr 2021 | | | | | |  |  |  |  |  |  |  |  |  |  |  |  |  |
| #12,"'virus'/exp OR virus*:ti,ab,kw OR viral:ti,ab,kw",1540286,10 Apr 2021 | | | | |  |  |  |  |  |  |  |  |  |  |  |  |  |  |
| #11,"'abscess'/exp OR abscess*:ti,ab,kw OR ((toxoplasmos$s NEAR/3 cerebr*):ti,ab,kw)",150790,10 Apr 2021 | | | | | | | |  |  |  |  |  |  |  |  |  |  |  |
| #10,"'urinary tract infection'/de OR ((urinary NEAR/3 infect*):ti,ab,kw)",130603,10 Apr 2021 | | | | | | |  |  |  |  |  |  |  |  |  |  |  |  |
| #9,"osteomyelit*:ti,ab,kw",30021,10 Apr 2021 | | |  |  |  |  |  |  |  |  |  |  |  |  |  |  |  |  |
| #8,"'ventilator associated pneumonia'/de OR 'ventilator associated pneumonia':ti,ab,kw",13126,10 Apr 2021 | | | | | | | |  |  |  |  |  |  |  |  |  |  |  |
| #7,"'cellulitis'/de OR cellulit*:ti,ab,kw OR phlegm$n:ti,ab,kw",28572,10 Apr 2021 | | | | | |  |  |  |  |  |  |  |  |  |  |  |  |  |
| #6,"mening*:ti,ab,kw OR endocardit*:ti,ab,kw",191663,10 Apr 2021 | | | | |  |  |  |  |  |  |  |  |  |  |  |  |  |  |
| #5,"'meningitis'/de OR 'endocarditis'/de OR 'bacterial endocarditis'/de",95596,10 Apr 2021 | | | | | | |  |  |  |  |  |  |  |  |  |  |  |  |
| #4,"((antifungal OR 'anti fungal' OR fungicides) NEAR/3 (therapeutic$ OR agent$ OR antibiotic$)):ti,ab,kw",19209,10 Apr 2021 | | | | | | | | | |  |  |  |  |  |  |  |  |  |
| #3,"'antifungal agent'/de",62828,10 Apr 2021 | | |  |  |  |  |  |  |  |  |  |  |  |  |  |  |  |  |
| #2,"'mycosis'/de OR mycos$s:ti,ab,kw",65458,10 Apr 2021 | | | |  |  |  |  |  |  |  |  |  |  |  |  |  |  |  |
| #1,"'sepsis'/exp OR endotoxemia$:ti,ab,kw OR fungemia$:ti,ab,kw OR candidemia$:ti,ab,kw OR ((endotoxin NEAR/1 shock$):ti,ab,kw) OR (((septic OR toxic) NEAR/1 shock$):ti,ab,kw) OR ((blood NEAR/1 poison*):ti,ab,kw) OR ((bloodstream NEAR/1 infection$):ti,ab,kw) OR py$emia$:ti,ab,kw OR pyohemia$:ti,ab,kw OR seps$s:ti,ab,kw OR septic*:ti,ab,kw OR uroseps$s:ti,ab,kw OR bacteremia:ti,ab,kw",388619,10 Apr 2021 | | | | | | | | | | | | | | | | | | |

**Results of CPG databases search**

- **CPG Databases:** GIN Library (n =1), ECRI Guidelines Trust (n=4), DynaMed (n=30), NICE (n=1), and SIGN (n=0)
- **Professional Societies:** AAP (n =1), CPS (n=0), RCPC (n=1), BAPM (n=0), SNS (n=0). Citation searching (n =0)

# Supplementary Figures and Tables

## *Supplement 2. Figure PRISMA 2020 flow diagram for new systematic reviews which included searches of databases, registers and other sources*


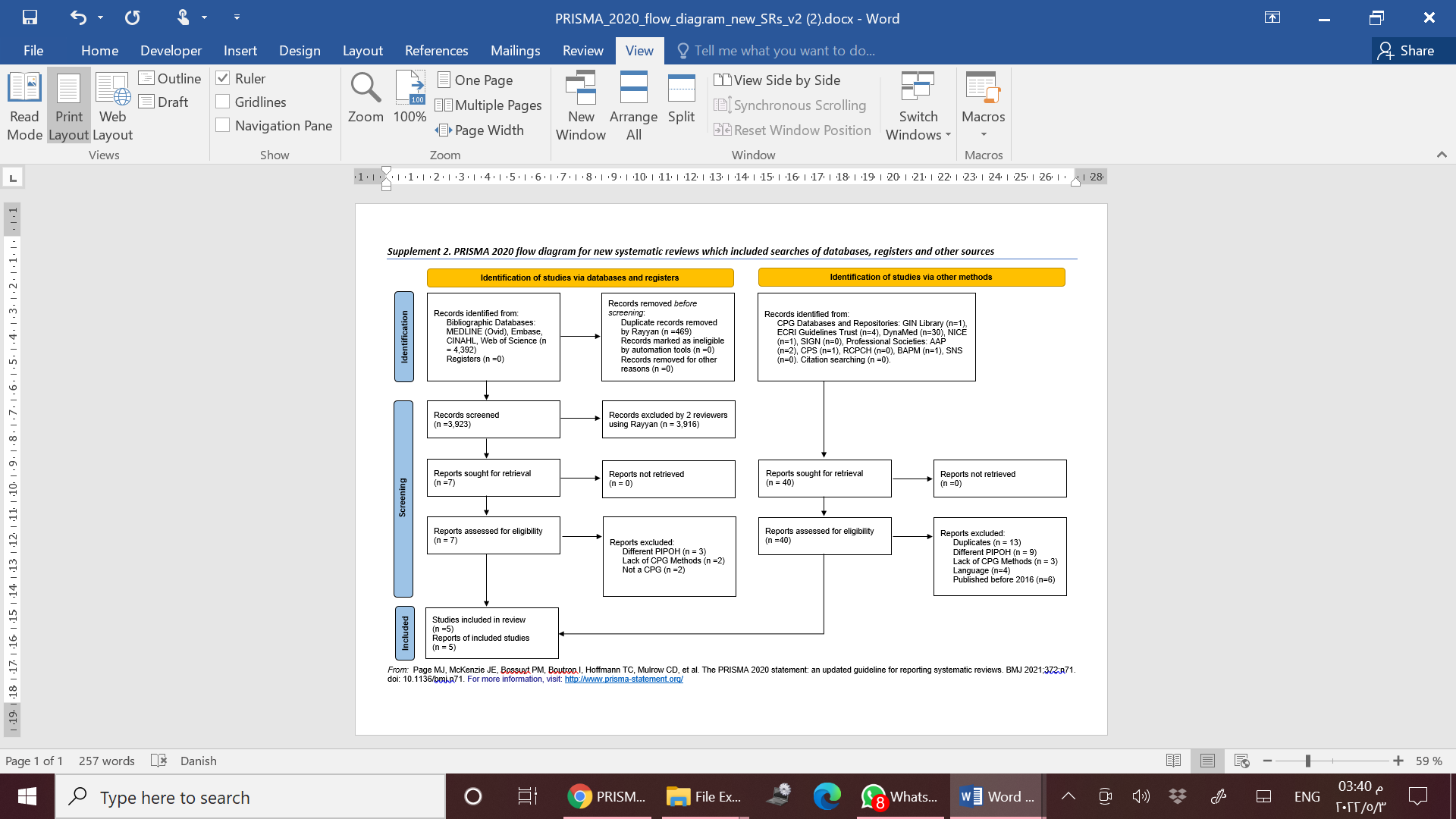


***From:*** Page MJ, McKenzie JE, Bossuyt PM, Boutron I, Hoffmann TC, Mulrow CD, et al. The PRISMA 2020 statement: an updated guideline for reporting systematic reviews. BMJ 2021;372:n71. doi: 10.1136/bmj.n71. For more information, visit: http://www.prisma-statement.org/

## *Supplement 3.1. Table for interpretation of the strength of agreement according to K value.*

| **Value of K** | **Strength of agreement** |
| --- | --- |
| < 0.20 | Poor |
| 0.21 - 0.30 | Fair |
| 0.31 - 0.40 | Moderate |
| 0.41 - 0.60 | Good |
| 0.61 - 0.80 | Very good |
| 0.81 – 1.00 | Excellent |

## *Supplement 3.2. Table for Classification of the strength of agreement among the four raters against the five clinical guidelines*

|  | **Poor** | **Fair** | **Good** | **Very good** | **Excellent** | **Sum of scores** | **Sum of OA 1 scores** | **Overall assessment (OA 1)** |
| --- | --- | --- | --- | --- | --- | --- | --- | --- |
| 1. **AAP 2018 American Academy of Pediatrics CPG** | 1 | 0 | 12 | 3 | 8 | 262 | 13 | Good |
| 1. **AAP2 2018 American Academy of Pediatrics CPG** | 0 | 0 | 9 | 9 | 6 | 273 | 12 | Good |
| 1. **CPS 2017 Canadian Paediatric Society CPG** | 0 | 0 | 10 | 11 | 3 | 288 | 13 | Good |
| 1. **NICE 2021 National Institute for Clinical Excellence CPG** | 0 | 0 | 2 | 4 | 18 | 786 | 35 | Excellent |
| 1. **QH 2020 Queensland Health CPG** | 2 | 0 | 14 | 7 | 1 | 478 | 22 | Good |
